# Development of Adaptive Point-Spread Function Estimation Method in Various Scintillation Detector Thickness for X-ray Imaging

**DOI:** 10.3390/s23198185

**Published:** 2023-09-30

**Authors:** Bo Kyung Cha, Youngjin Lee, Kyuseok Kim

**Affiliations:** 1 Precision Medical Device Research Center, Korea Electrotechnology Research Institute (KERI), 111, Hanggaul-ro, Sangnok-gu, Ansan-si 15588, Gyeonggi-do, Republic of Korea; bkcha@keri.re.kr; 2 Department of Radiological Science, College of Health Science, Gachon University, 191, Hambangmoe-ro, Yeonsu-gu, Incheon 21936, Republic of Korea; 3 Department of Biomedical Engineering, Eulji University, 553, Sanseong-daero, Sujeong-gu, Seongnam-si 13135, Gyeonggi-do, Republic of Korea

**Keywords:** image restoration, non-blind deconvolution, image quality assessment, scintillator thickness, adaptive point-spread function

## Abstract

An indirect conversion X-ray detector uses a scintillator that utilizes the proportionality of the intensity of incident radiation to the amount of visible light emitted. A thicker scintillator reduces the patient’s dose while decreasing the sharpness. A thin scintillator has an advantage in terms of sharpness; however, its noise component increases. Thus, the proposed method converts the spatial resolution of radiographic images acquired from a normal-thickness scintillation detector into a thin-thickness scintillation detector. Note that noise amplification and artifacts were minimized as much as possible after non-blind deconvolution. To accomplish this, the proposed algorithm estimates the optimal point-spread function (PSF) when the structural similarity index (SSIM) and feature similarity index (FSIM) are the most similar between thick and thin scintillator images. Simulation and experimental results demonstrate the viability of the proposed method. Moreover, the deconvolution images obtained using the proposed scheme show an effective image restoration method in terms of the human visible system compared to that of the traditional PSF measurement technique. Consequently, the proposed method is useful for restoring degraded images using the adaptive PSF while preventing noise amplification and artifacts and is effective in improving the image quality in the present X-ray imaging system.

## 1. Introduction

A flat-panel detector (FPD) is an important device in digital radiographic imaging systems. The FPD is located behind the object to be imaged in the direction of radiation from the X-ray and gamma-ray sources. As radiation penetrates an object, it converts the attenuated information into electrical signals and then converts them into digital signals for a subsequent real-time display [1]. The inner structures of the objects in the radiation imaging scanner are represented by the relative intensity of the signals captured by the pixel array owing to the difference in the X-ray attenuation coefficients of the inner structures. These electrical signals are converted into digital signal outputs using a readout integrated circuit (ROIC) and provided to a backend computer system [2].

Digital radiography (DR) using FPD has the advantages of fast image-acquisition speed and wide dynamic range compared to conventional film and computed tomography (CR) techniques [3]. The FPD has fast image acquisition, allowing real-time diagnostics. When the radiation reaches the FPD, it is immediately converted into an electrical signal and digital image. The FPD consists of a pixel array of sensing elements called pixels, and an electronic circuit that includes the ROIC and a gate driver. This reduces the image distortion caused by blurring owing to long exposure times and ensures an accurate diagnosis [4]. Moreover, FPD has a wide dynamic range under various illumination conditions. Combining a phosphor or scintillator and a backplane (pixel array) in an FPD results in high light sensitivity, which allows for the sensitive detection of radiation [5].

Depending on the method used to convert radiation into electrical signals, methods can be broadly classified as direct or indirect [6,7]. A direct conversion detector converts the energy deposited into electric charges using photoconductors or semiconductor materials, including amorphous selenium (a-Se), thallium bromide, and gadolinium compounds, using materials with low atomic numbers [8]. In contrast, an indirect conversion detector improves the photon-to-electric signal conversion ratio by inserting a scintillator (e.g., thallium-doped cesium iodide [CsI:Tl], gadolinium oxysulfide [Gadox], barium fluoride, or cadmium tungstate [CdWO_4_]) in the process of converting photons to electrical signals in the direct conversion detector [9,10]. The spatial resolution of the indirect conversion detector is restricted by the blurring of the scattered light within the scintillators compared to that of the direct conversion detector [11]. Swank [12] investigated the noise factor when the resultant signal was formed by integrating scintillation pulses. This depends on the shape of the pulse-height distribution in the X-ray phosphors. Therefore, direct conversion detectors have a relative advantage for high-resolution imaging, such as mammography [13,14], and it is helpful to improve the image quality by optimizing the scintillator thickness for optimal detection quantum efficiency (DQE) with noise reduction, although the resolution and sharpness are reduced [15,16].

In general, the finite focal spot size, detector pixel size, and magnification affect the acquisition of information by distorting the original signal [17,18], which can be expressed by Equation (1), assuming a linear shift-invariant system [19].

(1) $$g\left(m,n\right)=PSF\left(m,n\right)⊗⊗\;f\left(m,n\right)+N(m,n),$$

where m and n describe the orthogonal coordinate indices, g is the degraded image, and f is the clean image. f is recovered from g by deconvolving the point-spread function (PSF), which is referred to as image deconvolution. Here, the PSF is considered to be the result of the influence of the finite focal spot size and detector pixel size. The magnitude within the object is assumed to be the same when the thickness of the object is much smaller than the SDD. The ⊗⊗ represents the 2D convolution operator, and N is the noise component. Notably, the PSF is most important to restore the blurred image, and there are three main techniques in which PSF can be measured in radiographic images: the pinhole, slit, and edge phantom [20,21,22] methods. Here, the area in which radiation passes is required to be ϕ ~ 10 μm in pinhole and slit phantom. The radiographic images acquired by these devices contain a blurred component owing to finite system conditions. A profile that includes the pinhole and slit areas can be represented as a PSF, as shown in Equation (2):

(2) $$PSF\left(m,n\right)=\frac{1}{\sigma \sqrt{2\pi }}\exp\left(-\frac{m^{2}+n^{2}}{2\sigma^{2}}\right),$$

where σ denotes the standard deviation of the Gaussian distribution function. In particular, the slit method was tilted by approximately 1.5°–3° to improve the measurement accuracy through oversampling [23,24]. The edge phantom was constructed from a 1 mm Tungsten (W) foil plate fixed on a 3 mm thick lead plate in accordance with the IEC 62220-1-1:2015 protocol [25,26]. The line spread function (LSF) of the edge phantom image was calculated by differentiating the profile between the penetrated radiation regions and vice versa.

Once the PSF is obtained through the above process, the restored image can be obtained by deconvolution of the degraded image, g. An approach that uses a pre-measured PSF is called a non-blind method. The advantage of this method is that it is more likely to succeed in deconvolution because it uses the exact PSF of an acquisition system [27,28]. Traditionally, Richardson–Lucy and Wiener filters are classic deconvolution methods [29,30], and fast deconvolution is performed to obtain restored images with improved sharpness. However, these images suffer from ringing artifacts [27]. Image prior-based deconvolution methods, including non-local-based approaches [31,32], fields of experts [33,34], and patch-based priors [35,36], can effectively prevent artifacts. However, these methods rely on empirical information and suffer from highly non-convex optimization problems and high computational costs [37]. Recently, machine learning-based deconvolution methods [38,39] have shown outstanding performance in image restoration; moreover, when combined with existing non-blind methods, they overcome the limitations of real-world applications to limited training data [37,40].

Despite the existing effective non-blind deconvolution methods, the noise in Equation (1) is amplified when high-pass filtering is performed by deconvolution, and the problem of image quality degradation remains [41]. To overcome these problems, a deconvolution approach based on regularization terms can be used to perform high-pass filtering with PSF while minimizing noise amplification [42,43,44]. In particular, penalty terms based on total variation (TV) and total generalized variation (TGV) are very effective at improving the sharpness of noisy and blurry images [45,46]. However, TV- and TGV-based regulation-term methods often tend to produce staircase artifacts [47]. Another approach is the deconvolution method after the noise reduction process, where deconvolution is performed considering the total PSF, composed of blurring in the imaging system and smoothing of the noise reduction algorithm [48]. This method can minimize noise amplification during deconvolution by removing the noise. However, restoring the lost detailed information using noise reduction algorithms is difficult.

Therefore, this study aims to convert the resolution of radiographic images acquired with a normal-thickness scintillator to that of a thin scintillator. The proposed method extracts the PSF with the most similar image quality between a high-resolution image convolving various PSF and a low-resolution image. Thus, a high-resolution image with minimal noise amplification was obtained by deconvolution using the PSF obtained from a low-exposure image with a normal-thickness scintillator. Simulations and experiments are conducted to verify the viability of the proposed framework. The following sections briefly describe the proposed scheme and discuss the results.

## 2. Materials and Methods

### 2.1. Imaging System Performance

**Figure 1:**
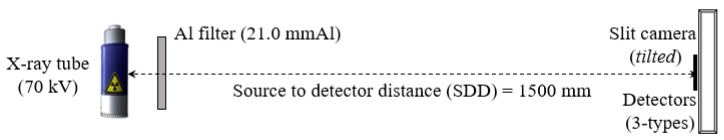
Simplified illustration of the exposure condition to measure the modulation transfer function (MTF) according to the scintillation thickness. Here, measurements were implemented in compliance with IEC 62220-1-1:2015 RQA-5 protocol.

**Figure 2:**
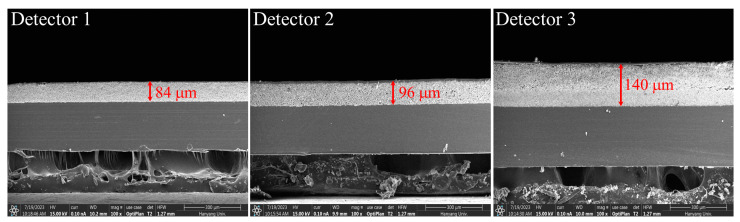
Scanning electron microscopy (SEM) images to measure each scintillator thickness.

**Figure 3:**
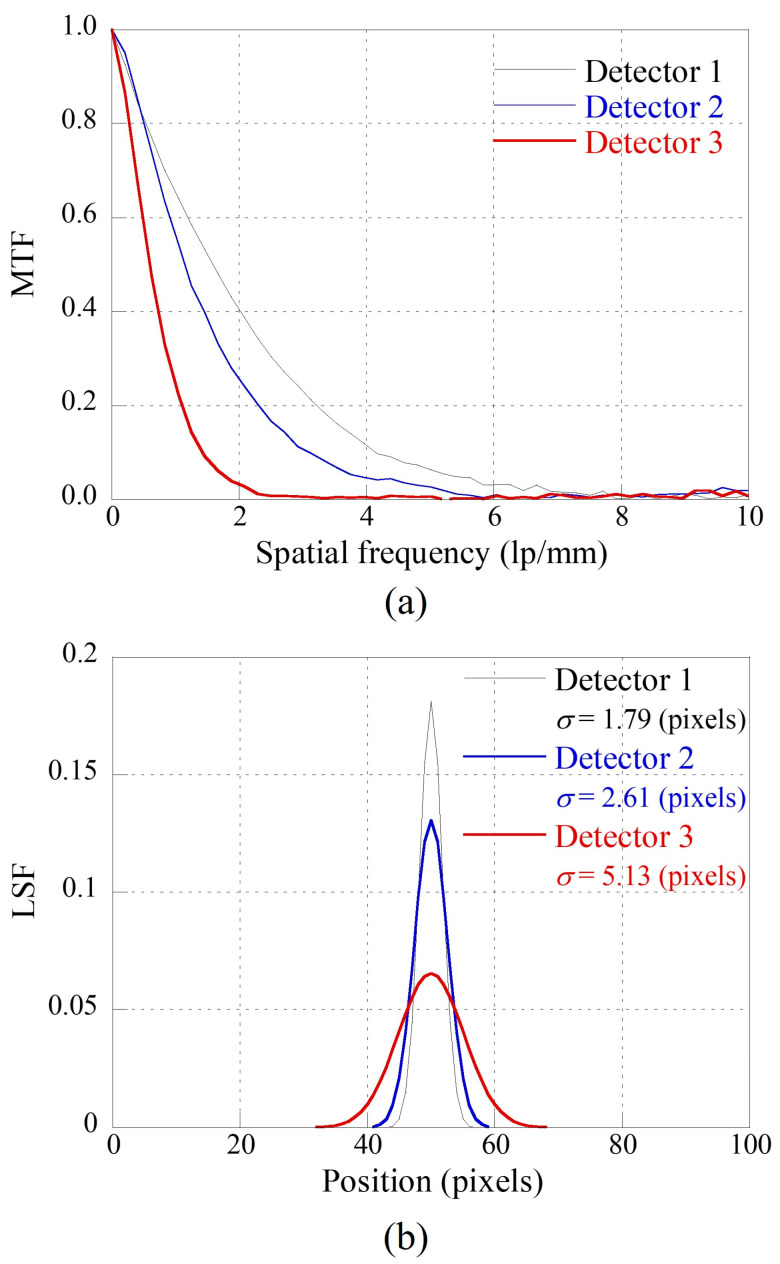
(**a**) MTF graphs of three detectors and (**b**) the calculated line spread functions (LSFs) based on Equations (2) and (3).

Figure 1 shows the exposure conditions for measuring the modulation transfer function (MTF) of the detector for each scintillator thickness. All the detectors were configured using a Gd_2_O_2_S:Tb scintillator and a CMOS image sensor (Rad-icon, Teledyne DALSA, California, USA). Here, Figure 2 shows the scanning electron microscope images (SEM) of the three detectors, and the detectors 1, 2, and 3 have scintillator thicknesses of 84 μm, 96 μm, and 140 μm, respectively. The scintillation thicknesses, pixel sizes, matrix sizes, and analog-to-digital conversion (ADC) resolutions of the detectors used in the experiments are summarized in Table 1. The experimental exposure conditions were documented following the guidelines of the IEC 62220-1-1:2015 protocol. According to the Radiation Quality (RQA)-5, the SDD was set to 150 cm, tube voltage to 70 kV, and additional filter of 21.0 mm Al. An X-ray tube (L10321, Hamamatsu Photonics K.K., Shizuoka-ken, Japan; focal spot size: 5 μm) was used to investigate the imaging system performance and conduct an experimental study. Figure 3a shows the MTF plots of all the detectors, where the *x*-axis is the spatial frequency. Radiographic images were acquired according to IEC 62220-1-1:2015 guidelines, while tilting a slit camera (07-24-1, Nuclear Associate Corp., Washington, USA) with a slit width of 10 μm to avoid aliasing [49]. MTF plots were calculated using Equation (3):

(3) $$MTF\left(f\right)=\left\lfloor F\{LSF\}\right\rfloor ,$$

where f is a 1D coordinate in the frequency domain, and F is an operator of the Fourier transformation. Figure 3b shows the LSF predicted by the inverse calculation of the measured MTF using Equations (2) and (3). The σ of the LSFs in Figure 3b were approximately 1.79 (pixels), 2.61 (pixels), and 5.13 (pixels) for detectors 1, 2, and 3, respectively.

Normalized noise power spectrum (NNPS) [50] representation is standard and useful for understanding the noise characteristics of an imaging system. This demonstrates the variation influenced by spatial frequency, indicating a limitation in utilizing the simulation for the design of degraded images based on Equation (1). Consequently, the analysis of the noise component was carried out using the Poisson–Gaussian mixture model as Equation (4), which has been recognized as the most suitable approach within the radiation degradation model due to its emphasis on noise characteristics [51,52].

(4) $$g\left(x\right)=PSF\left(x\right)⊗f\left(x\right)+\eta (x)\delta ,\;\eta^{2}\left(x\right)=\alpha x+\beta^{2},$$

where η(x) represents the standard deviation of the noise distribution at the pixel position x, which can be decomposed into Poisson noise, α, and the variance of Gaussian noise, $\beta^{2}$. Additionally, δ denotes zero-mean independent random noise with a standard deviation of one. Earlier research grounded in this model has substantiated its effectiveness [53,54]. Notably, Sutour et al. [55] anticipated noise parameters through non-parametric detection within uniform regions based on Kendall’s τ-coefficient [56,57]. This approach employs a robust polynomial noise level function (NLF) estimation method that relies on the correlation between the mean and variance observed in uniform regions.

**Figure 4:**
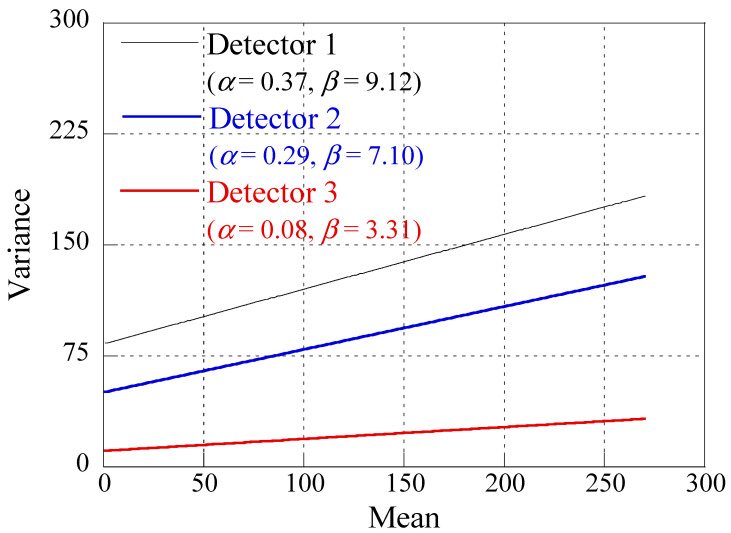
Predicted noise level function (NLF) of three detectors through the relationship between the mean and variance in homogeneous regions.

As illustrated in Figure 4, the NLF of the three detectors is depicted using the average of the white images, denoted as $\bar{S}$. The Poisson parameters, α, for detectors 1, 2, and 3 were approximately 0.37, 0.29, and 0.08, respectively. Moreover, their respective Gaussian parameters, β, were approximately 9.12, 7.10, and 3.31. These detector-specific PSF and noise parameter outcomes, derived from MTF and NLF measurements, were then employed in the simulation process to generate an image that authentically represents the underlying physical phenomena.

### 2.2. Data Acquisition

#### 2.2.1. Simulations

**Figure 5:**
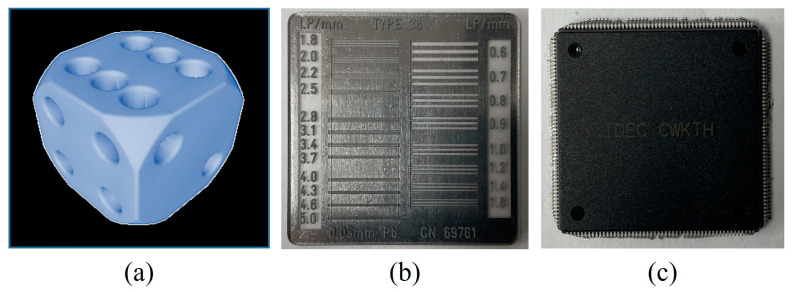
(**a**) A 3D numerical phantom in simulation, (**b**) a high-resolution line chart phantom, and (**c**) an electronic device in experiment.

Figure 5a shows a 3D numerical dice phantom used in the simulation. The geometrical acquisition conditions were the same as those of Section 2.1. The dice phantom has 300 × 300 × 300 voxels and the size of voxels is 0.1 × 0.1 × 0.1 mm^3^. In the simulation, a degradation image is created according to Equations (1) and (4) based on the previously calculated σ of PSF and α and β of noise parameters. To generate a noisy image, we used the *imnoise* (∙) and *poissrnd* (∙) functions in the MATLAB^TM^ (version 8.3) toolbox. The dice phantom composed of the methyl methacrylate (PMMA) and attenuation images were obtained based on the ray-tracing method. The SDD was set to 150 cm, the source-to-object (SOD) was set to 100 cm, and the tube voltage was set to 70 kV at mono-energy spectrum. Note that, the effect of scatter radiation is not considered for the proof of principle for Equation (4).

#### 2.2.2. Experiments

Figure 5b also shows a high-resolution line chart phantom (Type 38, CN 69761, Active Radsys, Italy) and Figure 5c an electronic device used in the experiment. Here, we used high-resolution patterns in the line chart, which had 20 groups from 0.6 to 5.0 lp/mm. This pattern is enclosed in plastic, which can be assumed to be a tissue-equivalent material. The line chart phantom and electronic device have thicknesses of 0.1 mm and 2 mm or less. The acquired signal was subjected to preprocessing, including dark analysis, image lag analysis, and a uniformity test to evaluate the image quality [22]. The specifications of the normal workstation were as follows: OS, Windows 10; CPU, Intel Core i7 10700; and RAM, 64 GB.

### 2.3. Proposed Restoration Framework Based on the Adaptive PSF Estimation

As mentioned, the resolution and amount of noise are tradeoffs according to the scintillator thickness when the exposure conditions and pixel size of the TFT panel are the same. In general, thick scintillation results in less noise and a higher resolution than thin scintillation. The goal of the proposed method is to restore the resolution from that of a thick scintillation detector to that of a thin scintillation detector while minimizing noise amplification. Algorithm 1 shows a simplified illustration of the proposed restoration framework based on suitable PSF estimation using the factors for image quality assessment.
**Algorithm 1** Structure of proposed scheme to estimate adaptive PSF**1:  Input**: Initial matrix *IMG*_1_, *IMG*_2_**2:  Output**: Complete matrix *FSF_σ_* **3:  Function***Initialize* (): **4:**    *Sigma_val_* = 0.01 to end (empirically); **5:**    Preallocation (*SSIM_val_*, *FSIM_val_*); **6:  END** **7:  Function**
*Main* (): **8:    For** val = *Sigma_val_* (start): *Sigma_val_* (end) **do** **9:**         *PSF_val_* ← Input sigma in Equation (2) (val); **10:**       *IMG*_1_*__blur_* = *IMG*_1_
⊗⊗ *PSF_val_*; **11:**       *SSIM_val_* (val) ← Calculate the Equation (5) (*IMG*_1_*__blur_*, *IMG*_2_); **12:**       *FSIM_val_* (val) ← Calculate the Equation (6) (*IMG*_1_*__blur_*, *IMG*_2_); **13:    END For** **14:**    *SSIM_σ_* = find the index (max (*SSIM_val_*)); **15:**    *FSIM_σ_* = find the index (max (*FSIM_val_*)); **16:**    *FSF_σ_* = average (*SSIM_σ_*, *FSIM_σ_*); **17: Return**
*FSF_σ_***18: END**

In brief, radiographic images were obtained using the scintillator thickness (here, we denote the thin scintillator thickness image as *IMG*_1_ and the thick scintillator thickness image as *IMG*_2_). Then, the *PSF_val_* was generated using one of a set of the sigma value (*Sigma_val_*) and blurred image (*IMG*_1_*__blur_*) designed when performing the convolution between *IMG*_1_ and *PSF_val_*. The structural similarity index (SSIM) and feature similarity index (FSIM) of *IMG*_1_*__blur_* and *IMG*_2_ were computed. The SSIM factor is considered to be correlated with image quality perception from the perspectives of loss of correlation, luminance distortion, and contrast distortion [58]. This factor can be defined by Equation (5):

$$SSIM={l\left(x,y\right)}^{\alpha }{c\left(x,y\right)}^{\beta }{s\left(x,y\right)}^{\gamma }=\frac{\left(2\mu_{x}\mu_{y}+C_{1}\right)}{\left(\mu_{x}^{2}+\mu_{y}^{2}+C_{1}\right)}\times \frac{\left(2\sigma_{xy}+C_{2}\right)}{\left(\sigma_{x}^{2}+\sigma_{y}^{2}+C_{2}\right)}\times \frac{\left(\sigma_{xy}+C_{3}\right)}{\left(\sigma_{xy}+C_{3}\right)},$$

(5) $$C_{1}={\left(k_{1}L\right)}^{2},C_{2}={\left(k_{2}L\right)}^{2},C_{3}=\frac{C_{2}}{2},$$

where $l\left(x,y\right)$, $c\left(x,y\right)$, and $s\left(x,y\right)$ are the luminance comparison function, contrast comparison function, and structure comparison function, respectively. $u_{x,y}$, $\sigma_{x,y}$, and $\sigma_{xy}$ are the local means, the standard deviations, and cross-covariance, respectively, of images x and y. C is a small positive constant (e.g., $k_{1}$ and $k_{2}$ are 0.01 and 0.03 as a default value, respectively), and L is the dynamic range. α, β, and γ are weight constants, and we set those constants to one in this study. In addition, by applying the default as $C_{3}=\frac{C_{2}}{2}$, the SSIM can be organized as $\frac{\left(2\mu_{x}\mu_{y}+C_{1}\right)\left(2\sigma_{xy}+C_{2}\right)}{\left(\mu_{x}^{2}+\mu_{y}^{2}+C_{1}\right)\left(\sigma_{x}^{2}+\sigma_{y}^{2}+C_{2}\right)}$. The closer to the SSIM value, the better the adaptive σ-value of the PSF. In addition, FSIM is also a full-reference image quality assessment, and it uses important low-dimensional properties such as edges and zero crossings to determine image quality [59]. FSIM uses phase congruency (PC) and gradient of magnitude (GM) maps. FSIM was defined as follows:

(6) $$FSIM=\frac{\sum_{x\in \Omega }{{[S}_{PC}\left(x\right)]}^{\alpha }\cdot {{[S}_{G}\left(x\right)]}^{\beta }\cdot PC(x)}{\sum_{x\in \Omega }PC(x)},S_{PC}\left(x\right)=\frac{2{PC}_{1}\left(x\right)\cdot {PC}_{2}\left(x\right)+T_{1}}{{PC}_{1}\left(x\right)+{PC}_{2}\left(x\right)+T_{1}},S_{G}\left(x\right)=\frac{2{GM}_{1}\left(x\right)\cdot {GM}_{2}\left(x\right)+T_{2}}{{{GM}_{1}}^{2}\left(x\right)+{{GM}_{2}}^{2}\left(x\right)+T_{2}},$$

where ${PC}_{1,2}$ and ${GM}_{1,2}$ represent the PC and GM maps of the input images, respectively. $T_{1}$ is a positive constant that controls the stability of $S_{PC}$ and $T_{2}$ is a positive constant according to the dynamic range of GM value. Ω denotes the whole image spatial domain, and α and β are balance parameters between PC and GM features. In this study, we followed the reference paper and used α=β=1. We found the index as the maximum value in the obtained SSIM (*SSIM_val_*) and FSIM (*FSIM_val_*) results. The optimal ${PSF}_{\sigma }$ can be used to calculate the average derived sigma index values. Finally, non-blind deconvolution was performed using the ${PSF}_{\sigma }$ as Equation (7):

(7) $$f*=\underset{{\mathrm{IMG}}_{2}\in Q}{\mathrm{argmin}}{\left\|{PSF}_{\sigma }⊗⊗{IMG}_{2}-{IMG}_{2}\right\|}_{2}^{2}+\lambda {\left\|\nabla {IMG}_{2}\right\|}_{1},$$

where Q is the set of feasible ${IMG}_{2}$, $\left\|{PSF}_{\sigma }⊗⊗{IMG}_{2}-{IMG}_{2}\right\|$ is the fidelity term, $\left\|\nabla {IMG}_{2}\right\|$ is the regularization term, and λ is the balancing factor due to control of the signal-to-noise ratio (i.e., λ=0.01 was used in this study). Here, the regularization term was set to the total variation-based *l*_1_-norm calculation to avoid artifacts [44,48]. The solving method to find the f* used the augmented Lagrangian of the problem technique [60], and the tolerance used $1\times 10^{-4}$ with an iteration loop stop condition.

### 2.4. Quantitative Evaluation of Image Performance

The profile, contrast-to-noise ratio (CNR) [61], and GM [59] were selected for quantitative image quality assessment. The CNR is commonly used to assess the distinguishability of an object or feature from background noise. GM is a metric used to formulate the sharpness of an image. The CNR and GM were calculated as follows:

(8) $$CNR\;value=\frac{\left|\mu_{T}-\mu_{B}\right|}{\sqrt{{\sigma_{T}}^{2}+{\sigma_{B}}^{2}}},$$

(9) $$GM\;value=\sqrt{{\left(I⨂f_{h}\right)}^{2}+{\left(I⨂f_{v}\right)}^{2}},$$

where $\mu_{T,B}$ and $\sigma_{T,B}$ denote the mean and standard deviation of the target or background region-of-interest (ROI), respectively. Generally, a higher CNR value indicates that it is easier to identify objects in an image by considering noise characteristics. 

I denotes the input image and $f_{h}$ and $f_{v}$ are gradient operators according to the horizontal and vertical directions, such as the Sobel, Canny, Prewitt, Scharr, Laplacian, and hybrid edge operators [62]. The closer the GM value of the initially calculated thin scintillator image, the more suitable the image restoration.

## 3. Results and Discussion

### 3.1. Simulations

**Figure 6:**
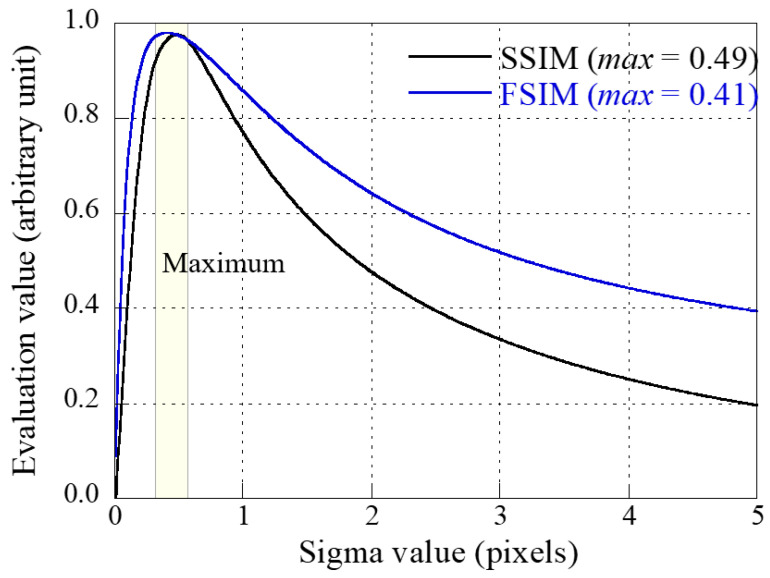
Graphs of SSIM and FSIM values between thick scintillator-based image and blurred thin scintillator-based image by convolving the PSF in the simulation.

Figure 6 shows the plots of the calculated SSIM and FSIM values between the thick scintillator-based simulated radiographic image, *IMG*_2_, and the thin scintillator-based blurred image by convolving the PSF *IMG*_1_*__blur_*. Here, the simulation image was designed using the numerical phantom shown in Figure 5a, and artifacts were added based on Equations (1) and (4) with the precalculated system performance evaluation parameters measured on the three detectors. The adaptive sigma for the PSF, determined by the highest SSIM value between detectors 1 and 2, was computed as 0.49 (pixels), while the corresponding value for the FSIM factor was approximately 0.41 (pixels). The average sigma across all values was calculated to be 0.45 (pixels). Similarly, the suitable sigma for detectors 3 and 1, determined through the same process, was approximately 0.63.

**Figure 7:**
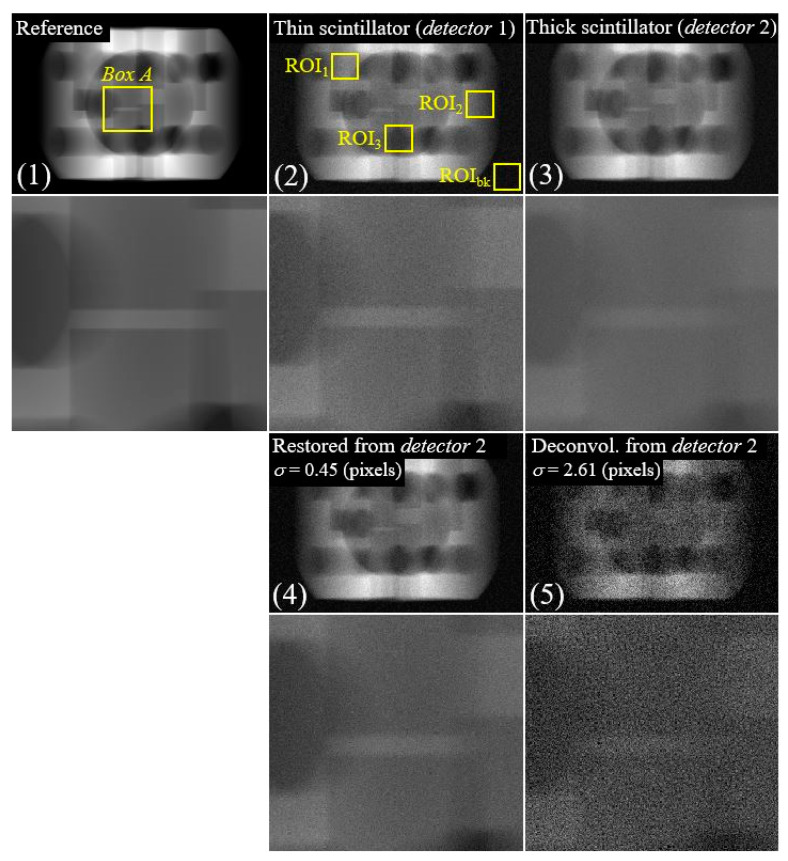
Simulation results illustrating: (1) the reference image; (2) the degraded image assuming a thin scintillator-based detector 1; (3) the degraded image assuming a thick scintillator-based detector 2; (4) the restored image obtained through simulation using the proposed PSF for detector 2; (5) deconvolution image derived from the simulated image of detector 2 using an alternative PSF.

Figure 7 illustrates various image scenarios: (1) the reference image; (2) a degraded image assuming a thin scintillator-based detector 1; (3) a degraded image assuming a thick scintillator-based detector 2; (4) a restored image from detector 2 using the proposed PSF (*σ* = 0.45 pixels); (5) deconvolution images from detector 2 employing alternative PSF (*σ* = 2.61 pixels for Detector 1). Notably, the non-blind deconvolution based on the proposed PSF demonstrates effective resolution restoration while minimizing noise amplification, emphasizing its utility for thin scintillator-based simulated image restoration.

**Figure 8:**
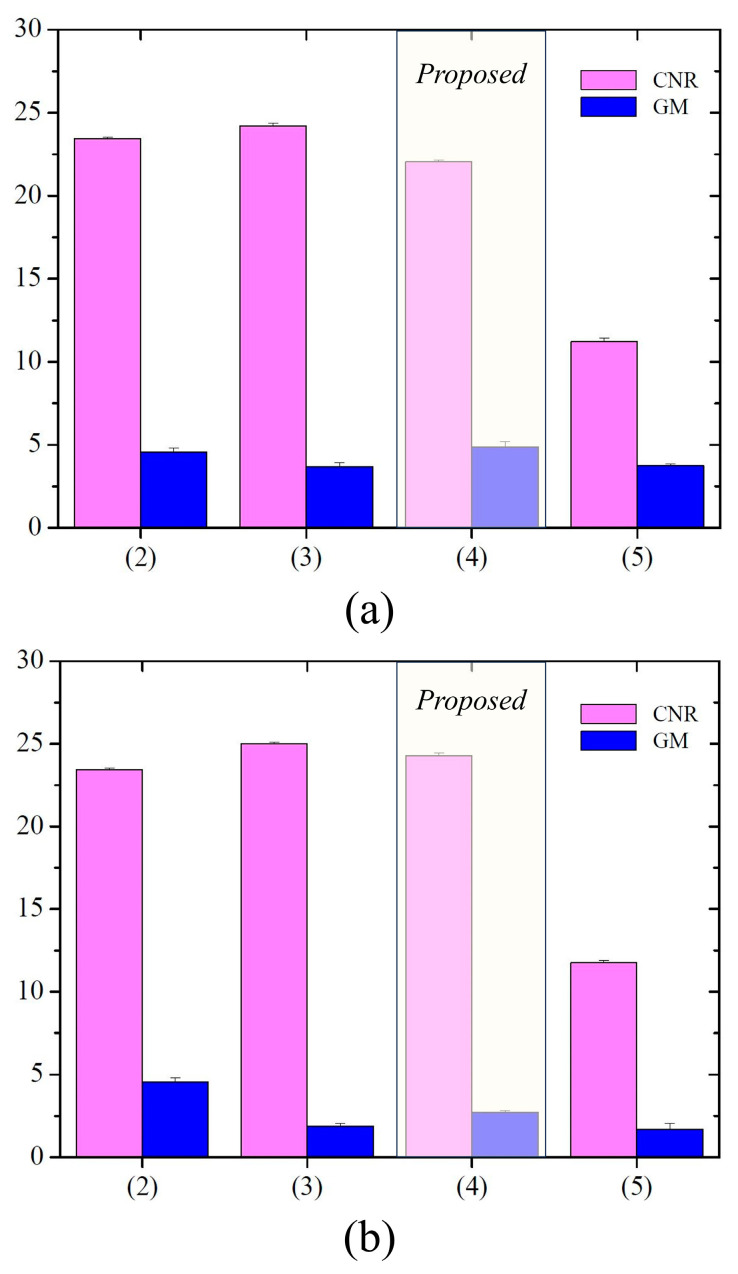
Contrast-to-noise ratio (CNR) and gradient of magnitude (GM) bar graphs; (**a**) the results using the simulation images of detector 1 and detector 2, and (**b**) those of detector 1 and detector 3.

Figure 8 presents CNR and GM results for degraded and restored images using degradation parameters from three detectors: (a) results employing simulation images of detectors 1 and 2, and (b) results using detectors 1 and 3. Here, the CNR value is calculated and averaged using the values within the three ROIs depicted in the thin scintillator image (2) of Figure 7. In Figure 8a, average CNR values are approximately 23.43, 24.21, 22.04, and 11.21 for degraded images with detectors 1 (2), detector 2 (3), proposed image from detector 2 (4), and deconvolution images using *σ* = 2.61 pixels (5), respectively. Similarly, in Figure 8b, the average CNR values are approximately 23.43, 25.01, 24.28, and 11.74 for degraded images with detectors 1 (2) and 3 (3), proposed image from detector 3 (4), and deconvolution images using *σ* = 5.13 pixels (5), respectively. The GM values in Figure 8a,b were approximately 3.68, 4.57, 4.88, and 3.35, and 1.88, 4.57, 2.73, and 1.54, respectively. These results indicate that the resolution of the proposed image was improved compared with those of images (2) and (5). A quantitative assessment of the high-resolution image derived from the degraded image, which assumes the thick scintillator-based detector 2 (3), further validates a satisfactory resolution enhancement. Note that, utilizing the suitable PSF during non-blind deconvolution achieves resolution improvement and effectively curbs noise amplification in the resulting image (5).

### 3.2. Experiments

**Figure 9:**
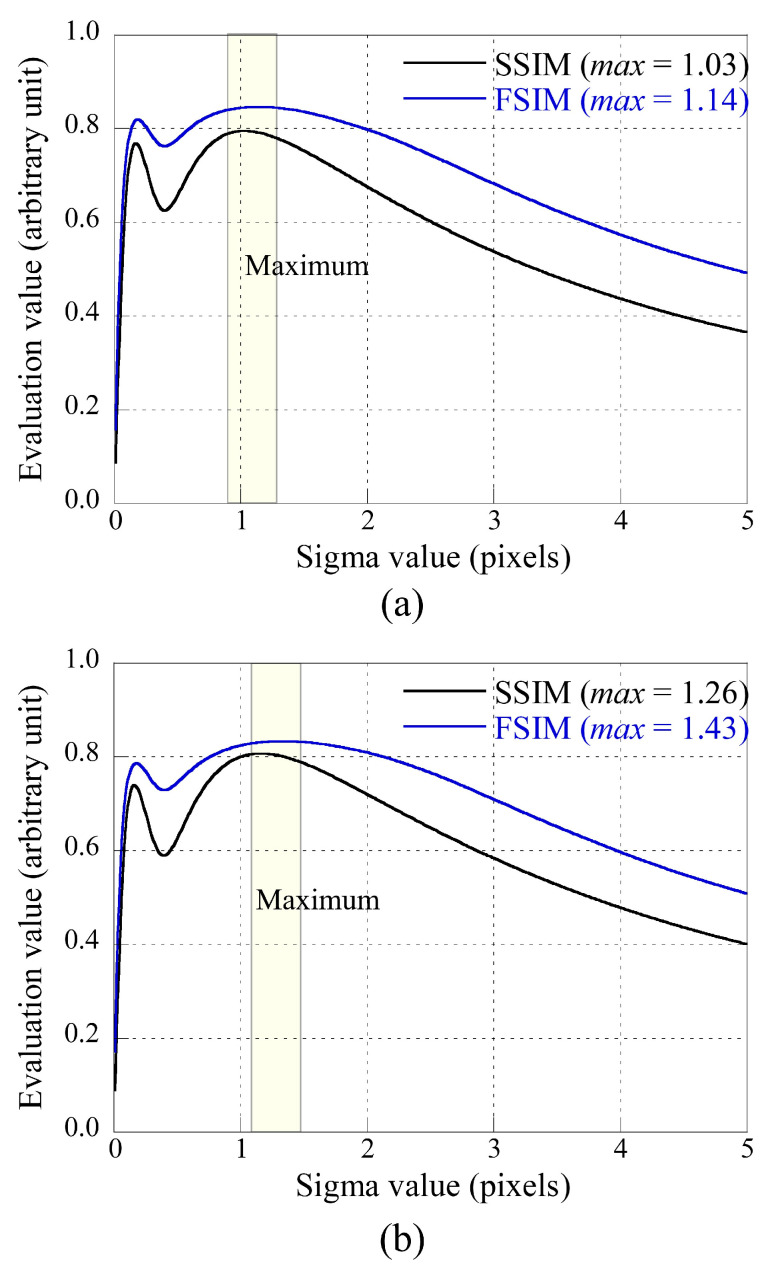
Plots of the SSIM and FSIM values; (**a**) detectors 1 and 2, and (**b**) detectors 1 and 3.

Figure 9 shows the plots of the SSIM and FSIM values used to calculate the adaptive sigma value of the PSF. Here, (a) compares the experimental images (*IMG*_2_ and *IMG*_1_*__blur_*) of detectors 1 and 2, and (b) compares those of detectors 1 and 3. The maximum values of SSIM and FSIM were predicted for sigma values of (a) 1.03 and 1.14, and (b) 1.26 and 1.43, respectively. The average sigma value was calculated as 1.09 between detector 1 and 2, and 1.35 between detector 1 and 3. Here, we can see that the first peak occurs around 0.4 pixels and then the SSIM and FSIM values decrease and then increase again. This is a different tendency compared to that of the simulation result in Figure 6, and this is expected to be caused by asymmetric PSF, various noises, and physical distortions such as scatter radiation.

**Figure 10:**
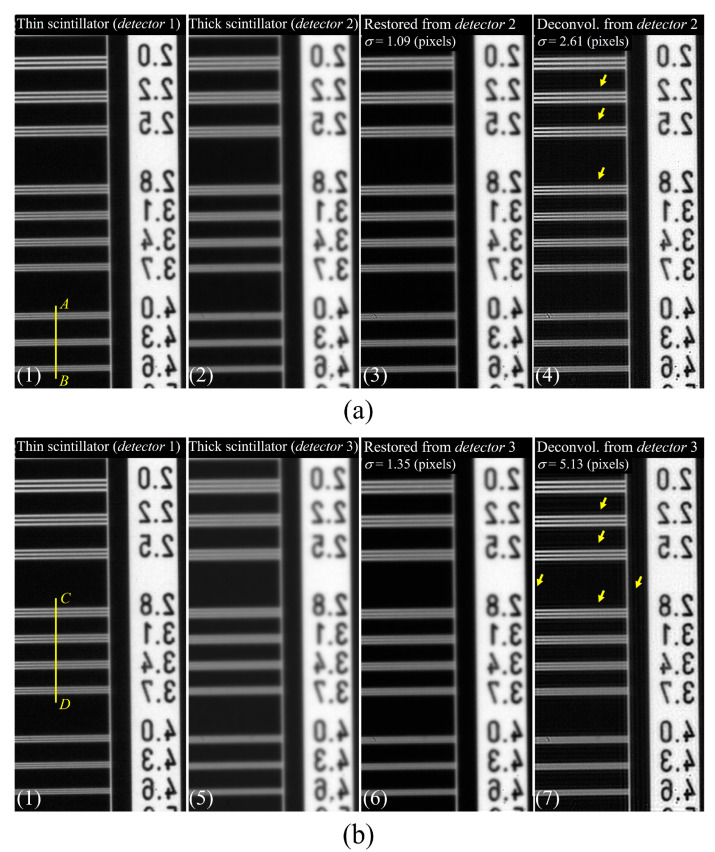
Experimental results of the radiographic image with a thin scintillator for detector 1, the degraded image with a thick scintillator, the proposed image derived from the thick scintillator image, and the deconvolution images utilizing the measured PSF to evaluate system performance. Here, (**a**) represents the outcomes between detectors 1 and 2, and (**b**) similarly depicts the outcomes between detectors 1 and 3.

Figure 10 presents illustrative instances, including a radiographic image captured with a thin scintillator in detector 1, a degraded image attained with a thick scintillator, the resultant image using the proposed approach from a thick scintillator image, and deconvolution images employing the measured PSF for assessing system performance. Specifically, (a) showcases outcomes between detectors 1 and 2, while (b) delineates outcomes between detectors 1 and 3. The application of the proposed framework for image restoration enhances overall image quality concerning the resolution and noise characteristics in contrast to radiographic images captured with a thin scintillator (detector 1) and thick scintillators (detectors 2 and 3). These findings underscore the significance of deriving the optimal PSF by considering comprehensive quantitative evaluation criteria. Moreover, the deconvolution results for a PSF corresponding to system performance (σ = 2.61 pixels and 5.13 pixels in detectors 2 and 3, respectively) considerably improved the resolution compared with the proposed method. However, it has limitations in that noise is significantly amplified, and artifacts may occur, as indicated by the yellow arrows, owing to excessive high-frequency filtering. These results indicate that the optimal PSF is derived by considering the quantitative evaluation factors.

**Figure 11:**
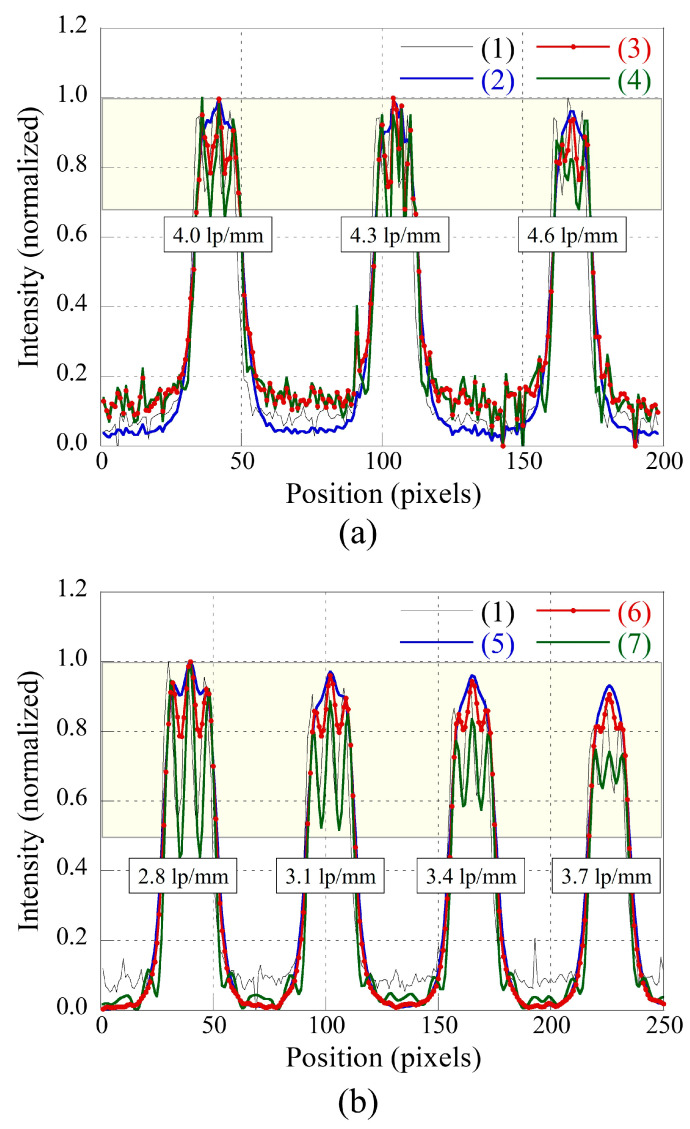
Normalized intensity profiles as measured along (**a**) line *AB* and (**b**) line *CD* are indicated in Figure 10a and Figure 10b, respectively.

Figure 11 presents the 1D normalized intensity profiles measured along (a) the line *AB* and (b) line *CD*, as indicated in Figure 10a and Figure 10b, respectively. The intensity profile of the restored image, acquired using the proposed method, exhibits differentiation between 2.0 lp/mm and 4.6 lp/mm. Noticeable improvement in resolution is apparent when compared to the thick scintillator images ((2) and (5)). Clear separation in the line bars is also evident when contrasted with the thin scintillator image (1), except for the 3.7 lp/mm case in Figure 11b. At 3.7 lp/mm, the profile result of the proposed restored image does not distinctly differentiate from the profiles of (1) and (7). However, this can be improved by increasing the sigma size of the PSF, although it may result in noise amplification.

**Figure 12:**
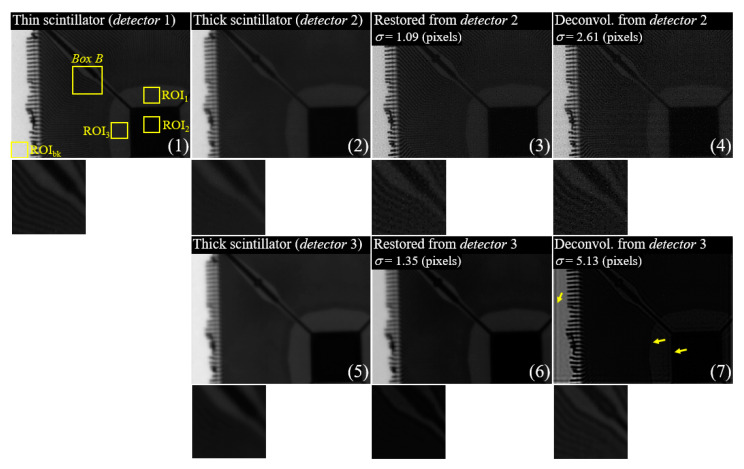
Examples of electronic device images, a restored image using the proposed method, and a deconvolution image using the PSF obtained by measuring the system performance. Here, the images of (1) to (4) **a** show the results using detectors 1 and 2, and the images of (1) and (5) to (7) show results using detectors 1 and 3.

Figure 12 shows the experimental results of an electronic device radiographic image, a restored image using the proposed approach, and a deconvoluted image using the PSF of the imaging system performance. Here, (a) shows the results obtained using detectors 1 and 2, and (b) shows the results obtained using detectors 1 and 3. The magnified image shows a zoomed-in view of Box *B* in Figure 12.

Figure 13 shows the results of the CNR and GM with the ROIs in Figure 12. The average CNR values in Figure 13a,b were calculated approximately as 29.04, 32.51, 26.59, and 22.37 for images (1) to (4), and 34.75, 31.41, and 6.13 for images (5) to (7). Moreover, the average GM values in Figure 13a,b were determined to be approximately 5.70, 3.59, 5.33, and 5.64 for images (1) to (4), and 1.30, 4.53, and 4.84 for images (5) to (7).

**Figure 14:**
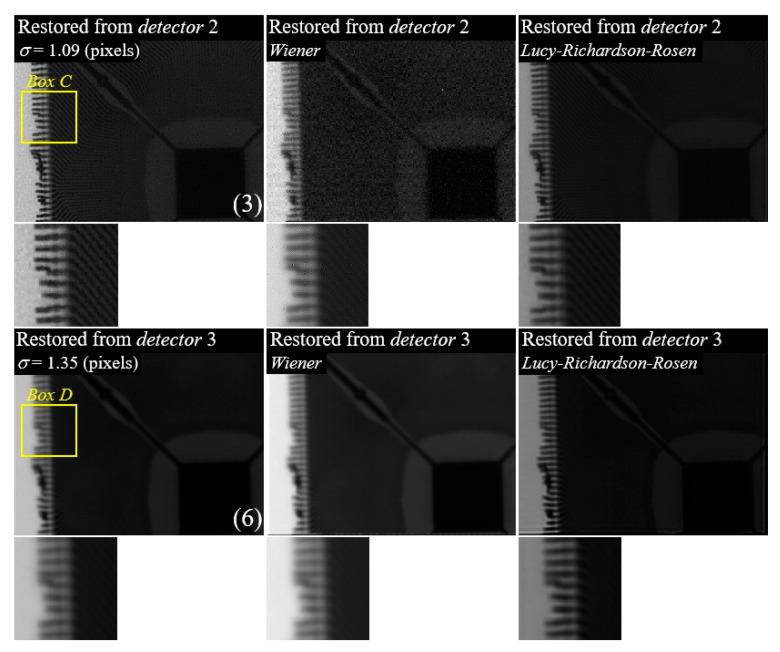
Experimental results of the iterative restoration method using regularization term-based minimization, a restoration method based on the Wiener filter, and the Lucy–Richardson–Rosen method. Here, the results above use detector 2 and another result below uses detector 3.

The purpose of the proposed method is to find the optimal PSF considering the human visible system, and then the best results can be achieved by applying a contextualized image restoration algorithm. Figure 14 shows the experimental results of the iterative restoration method using a regularization term, a restoration method based on the Wiener filter, and the Lucy–Richardson–Rosen method [29,30,63]. Here, the results above use detector 2 and another result below uses detector 3. Wiener filtering, also known as minimum mean-square-error filtering, takes into account the stochastic characteristic of blur and noise. It is reconstructed by Equation (10):

(10) $$\hat{F}=\left[\frac{H*}{{\left|H\right|}^{2}+K}\right]G,$$

where $\hat{F}$ is restored by the Wiener filter using the degraded image, G, in frequency domain, H is the PSF component in frequency domain, and H* is complex conjugate of H. K is the Wiener constant, which is related to the signal-to-noise ratio. The Wiener filter approximates inverse filtering by directly dividing the PSF when there are few noise components in the image. In contrast, the Wiener filter approaches zero and becomes similar to a frequency-rejection filter in a noisy image which has very large noise compared to the original image. The Lucy–Richardson–Rosen method replaces the correlation in the Lucy–Richardson algorithm by the non-linear reconstruction method [63]. The iterative Lucy–Richardson algorithm is calculated by Equation (11):

(11) $$\hat{g}(n+1)=\hat{g}(n)\left[\frac{f(n)}{\hat{g}(n)⊗PSF}⊗PSF^{\prime }\right],$$

where $PSF^{\prime }$ denotes the complex conjugate of PSF, and f(n) is the initial guess. f(n+1) is generated by convolving between PSF and f(n). The ratio between f(n+1) and f(n) is correlated with the PSF. This correlation is replaced by the non-linear reconstruction method, and the resulting residual is multiplied by the previous guess until the mismatch between the two metrics is within tolerance. This algorithm is also based on inverse filtering, and noise estimation is important. Therefore, it is essential that the noise characteristics are well derived and optimized for image restoration. These characteristics are demonstrated in Figure 14. The image from detector 2 has relatively more noise components, compared to that of the image from detector 3 at the same radiation dose. Therefore, the higher resolution image of the regularization method can be seen while optimizing the relative noise component, compared to that of the wiener filter and the Lucy–Richardson algorithm. However, when reconstructing the image of detector 3, which is less noisy, the result is not an optimal image owing to the obstacle of the regularization term of obtaining the high resolution. Comparatively, the Lucy–Richardson–Rosen method, which optimizes for noise characteristics, results in a much higher resolution. Therefore, the PSF obtained through the proposed method can achieve the best restoration result when deconvolution is performed by selecting the optimal reconstruction method according to the characteristics of the image.

In summary, the proposed method is considerably more effective in restoring the image by considering the overall image quality and predicting the appropriate PSF. However, this study had several limitations. First, some noise components are emphasized in high-pass filtering. Although the optimal PSF was predicted by accounting for noise amplification, high-frequency filtering inevitably introduces noise amplification. Several noise reduction approaches offer solutions to this problem. Chen et al. introduced an object function to solve for an optimal noise reduction image using an approximate TV regularization term and the weight of the fidelity term [64]. This model demonstrates that noise can be removed while preserving details and edges. Another approach for optimizing the parameters in the noise reduction algorithm is presented. Seo et al. optimized the similarity and weight parameters for a fast non-local means algorithm in low-dose computed tomography [65]. The optimal parameters of the noise reduction algorithm considering the X-ray exposure conditions yielded improved image quality in both quantitative and qualitative measurements. We believe that this method can be applied to reduce the noise component while maintaining sharpness and that it can significantly compensate for the problems of the proposed method.

Another limitation is the radiation–scatter component. The proposed method performs image restoration using the degraded image assumed in Equations (1) and (4). However, there is no contribution from the scatter component images in the assumed model, which have a significant impact on low-contrast radiographic image [66]. When X-rays penetrate an object, the transmitted radiation consists of primary and scattered components. The scattered component depends on the radiation energy, the field of view, and the thickness of the object [67,68]. Many studies on degradation models based on radiation–scatter characteristics have been published. The key idea behind these models is that the scatter component is modeled by adding or convolving primary radiation, and the scatter signal is mainly distributed at low frequencies [69,70,71]. Therefore, the low-frequency region contains various types of degradation information, including the effect on the scintillation thickness, and quantitative image analysis is required for an accurate image performance evaluation of the proposed algorithm. In continuous R&D, we plan to investigate the impact of the proposed method on scatter radiation using Monte Carlo simulations to describe a real radiographic imaging system.

## 4. Conclusions

The proposed framework predicts an adaptive PSF to convert an image from a normal-thickness scintillator to a thin scintillator. The main objective is to suppress noise amplification and artifacts as much as possible after deconvolution using adaptive PSF. The experimental results show that the average CNR value for the proposed image was approximately 31.41, which is approximately 1.08 times larger than that of the thin scintillator-based detector 1 image. The GM value of the proposed image was also approximately 4.53, which was approximately 3.48 times higher than that of the thick scintillator-based detector 3 images. These results indicate that the proposed method effectively solves the tradeoff problem between improving sharpness and preventing noise amplification. In conclusion, the proposed software is expected to be applicable to radiographic systems.

## Figures and Tables

**Figure 13:**
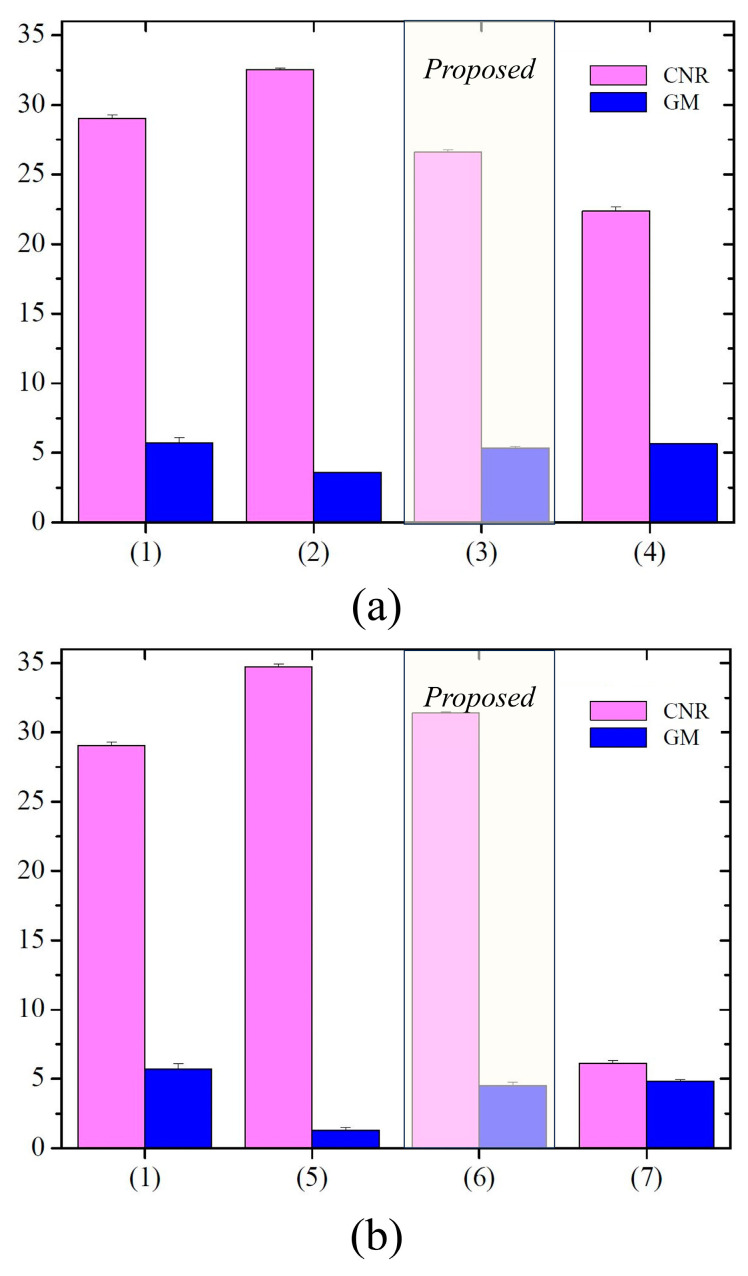
Bar graphs of CNR and GM results with ROIs in Figure 12. Here, (**a**) compares detector 1 and 2, and (**b**) compares detector 1 and 3.

**Table 1:** Detector specifications.

Name	Scintillator Thickness (μm)	Pixel Size (μm)	Pixel Matrix (Pixels)	ADC	σ of LSF (Pixels)
Detector 1	84	48	512 × 1024	12-bit	1.79
Detector 2	96	48	512 × 1024	12-bit	2.61
Detector 3	140	48	512 × 1024	12-bit	5.13

## Data Availability

Not applicable.
